# Genetic and Pharmacological Inhibition of GCN2 Ameliorates Hyperglycemia and Insulin Resistance in Type 2 Diabetic Mice

**DOI:** 10.3390/antiox11081584

**Published:** 2022-08-16

**Authors:** Juntao Yuan, Fang Li, Xiyue Shen, Junling Gao, Zhuoran Yu, Kai Luo, Bingqing Cui, Zhongbing Lu

**Affiliations:** College of Life Science, University of Chinese Academy of Sciences, Beijing 100049, China

**Keywords:** type 2 diabetes, GCN2, hyperglycemia, insulin resistance, hepatic steatosis, oxidative stress

## Abstract

It is well recognized that there is a strong and complex association between nonalcoholic fatty liver disease (NAFLD) and type 2 diabetes (T2D). We previously demonstrated that genetic knockout or pharmacological inhibition of general control nondepressible kinase 2 (GCN2), a well-known amino acid sensor, alleviated hepatic steatosis and insulin resistance in obese mice. However, whether GCN2 affects the development of T2D remains unclear. After a high-fat diet (HFD) plus low-dose streptozotocin (STZ) treatments, *Gcn2*^−/−^ mice developed less hyperglycemia, insulin resistance, hepatic steatosis, and oxidative stress than wild-type (WT) mice. Inhibition of GCN2 by intraperitoneal injection of 3 mg/kg GCN2iB (a specific inhibitor of GCN2) every other day for 6 weeks also ameliorated hyperglycemia, insulin resistance, hepatic steatosis, and oxidative stress in HFD/STZ- and leptin receptor deletion (db/db)-induced T2D mice. Moreover, depletion of hepatic GCN2 in db/db mice by tail vein injection of an AAV8-sh*Gcn2* vector resulted in similar improvement in those metabolic disorders. The protective mechanism of GCN2 inhibition in T2D mice was associated with regulation of the glucose metabolic pathway, repression of lipogenesis genes, and activation of the Nrf2 pathway. Together, our data provide evidence that strategies to inhibit hepatic GCN2 activity may be novel approaches for T2D therapy.

## 1. Introduction

The prevalence of type 2 diabetes mellitus (T2DM) in China has become a major threat to public health in recent decades. T2DM mainly results from being overweight and sedentary and is characterized by hyperglycemia and insulin resistance in target organs, including the liver, muscle, and kidney [1]. The liver is an essential organ for the maintenance of systemic glucose homeostasis. Drugs repressing hepatic glucose production, especially gluconeogenesis, are common treatments for patients with T2DM [2,3,4]. In addition, growing epidemiological evidence suggests a strong and complex association between T2D and nonalcoholic fatty liver disease (NAFLD) [5]. Excessive lipid deposition in the liver compromises hepatic insulin sensitivity and the control of glucose production, thereby accelerating the progression of T2D [6].

GCN2, a kinase of eukaryotic initiation factor 2α (eIF2α), is important in maintaining amino acid homeostasis under amino acid starvation conditions [7,8]. GCN2 is also involved in regulating hepatic gluconeogenesis and insulin sensitivity in response to fasting or leucine deprivation [9,10,11]. Interestingly, a recent report showed that upregulation of hepatic GCN2 caused by intermittent leucine deprivation contributes to long-lasting improvements in insulin sensitivity in high fat diet (HFD)-fed mice and diabetic mice (db/db) [10]. We previously demonstrated that deletion of *Gcn2* decreased blood glucose levels and increased insulin sensitivity in HFD-fed mice [12]. *Gcn2* deficiency also decreased blood glucose levels and improved cardiac dysfunction in T2D mice [13]. Moreover, we recently showed that pharmacologic inhibition of GCN2 ameliorated hepatic steatosis and insulin resistance in ob/ob and HFD-fed mice [14]. Therefore, we hypothesized that inhibition of GCN2 is a potential approach for T2DM therapy. In this study, we used *Gcn2*^−/−^ mice, AAV8-sh*Gcn2,* and the GCN2-specific inhibitor GCN2iB to investigate the effect of GCN2 on glucolipid metabolism in T2D mice.

## 2. Materials and Methods

### 2.1. Reagents and Antibodies

Streptozotocin (STZ), dihydroethidium (DHE), and oil red O were obtained from Sigma-Aldrich Co. LLC (#S0130, #D7008, and #O0625, St. Louis, MO, USA). 3-nitrotyrosine (3′-NT) and 4-hydroxynonenal (4-HNE) ELISA kits were purchased from Abcam (#ab116691 and #ab238538, Cambridge, UK). Protease and phosphatase inhibitor cocktails were obtained from Roche (#04693124001, #4906837001, Basel, Switzerland). GCN2iB was purchased from MedChemExpress LLC (#HY-112654, Princeton, NJ, USA). Leptin receptor-deficient mice (db/db) and HFD were obtained from HFK Bioscience Co. (Beijing, China). Recombinant adeno-associated virus serotype 8 (AAV8)-GFP and AAV8-sh*Gcn2* (targeting sequence: 5′-GGTATACAATGCTTTGGAA-3′) were obtained from Vigene Biosciences. Inc. (Shandong, China).

### 2.2. Experimental Animals

To induce T2D, 10-week-old male *Gcn2*^−/−^ mice and wild-type (WT) littermates were fed HFD (60% fat) for 8 weeks. Then, the mice were fasted for 6 h and given a single intraperitoneal injection of freshly prepared STZ (100 mg/kg, formulated in 0.1 M citrate buffer, pH 4.5). After six weeks, the mice were euthanized.

To inhibit GCN2 activity, db/db and HFD plus STZ-induced type 2 diabetic mice were treated with 3 mg/kg GCN2iB every other day via intraperitoneal injection for 6 weeks. At the end of the experiments, the mice were euthanized for subsequent experiments. To deplete hepatic *Gcn2*, db/db mice were treated with AAV8-sh*Gcn2* (1.1 × 10^12^ vg/per mouse) via tail vein injection. Mice were euthanized after 4 weeks.

Serum triglyceride (TG), alanine aminotransferase (ALT), and aspartate aminotransferase (AST) levels were measured using commercial kits from Nanjing Jiancheng Bioengineering Institute (#A110–2, #C009–2, and #C010–2, Jiangsu, China). Serum insulin levels were determined using an ELISA kit from ALPCO Diagnostics (#80-INSMSU-E01, Salem, NH, USA). Liver TG levels were measured using a kit from Applygen Inc. (#E1013, Beijing, China).

The oral glucose tolerance test (OGTT) was performed using an oral gavage of 2 g/kg glucose after overnight fasting. The insulin tolerance test (ITT) was performed via intraperitoneal injection of 0.75 U/kg insulin after fasting for 4 h. Approximately 10 μL of blood was obtained from a tail cut and was assessed for glucose levels using an Accu-Chek^®^ glucometer (Roche Diagnostics, Indianapolis, IN, USA).

During the experimental period, mice had free access to distilled water and commercial mouse chow. They were housed in individually ventilated cage systems under controlled temperature (22 ± 2 °C) and relative humidity (40–60%) conditions with a 12 h light/dark cycle. Protocols for animal experiments were carried out in accordance with the guide for the care and use of laboratory animals (Eighth edition, 2011).

### 2.3. Histopathology Staining

Liver paraffin sections (5 μm) were stained with hematoxylin and eosin (H&E). To assess hepatic steatosis and oxidative stress, frozen liver sections (4 μm) were stained with oil red O and DHE, respectively. At least 5 mice per group were used for these experiments.

### 2.4. Western Blot Analysis and Quantitative Real-Time Polymerase Chain Reaction (qPCR) Analysis

Liver tissues were homogenized with ice-cold lysis buffer (150 mM NaCl, 50 mM Tris-Cl, 1% Triton X-100, protease, and phosphatase inhibitor cocktail, and 100 μg/mL phenylmethylsulfonyl fluoride). The lysates were centrifuged at 12,000× *g* at 4 °C for 20 min, and then the supernatant was collected for subsequent experiments. Equal amounts of protein samples were loaded and separated by SDS-PAGE gels. The detailed protocol for western blot analysis was carried out as described previously [15,16]. Detailed information on the antibodies is listed in Table S1.

To extract total RNA, liver tissues were homogenized with TRIzol reagent. To obtain cDNA, the extracted RNA was subjected to reverse transcription reactions, which were performed using PrimeScript RT reagent kits (#RR036B, TaKaRa, Otsu, Japan). SYBR^®^ Premix ExTaqTM II kits (#RR820DS, TaKaRa, Otsu, Japan) were used for quantitative PCR. The primers used in this study are listed in Table S2. The mRNA levels of 18S ribosomal RNA were used as internal controls. The 2^−ΔΔCT^ method was used for qPCR data analysis.

### 2.5. Data and Statistical Analysis

Data are expressed as the means ± SD. GraphPad Prism 8 (GraphPad Software Inc., San Diego, CA, USA) was used for data analysis. Statistical significance between groups was determined by unpaired 2-tailed t test or one-way ANOVA followed by Fisher’s least significant difference test. *p* < 0.05 was defined as statistically significant.

## 3. Results

### 3.1. GCN2 Deficiency Ameliorates Hyperglycemia, Liver Dysfunction, and Insulin Resistance in HFD/STZ-Induced T2D Mice

**Figure 1:**
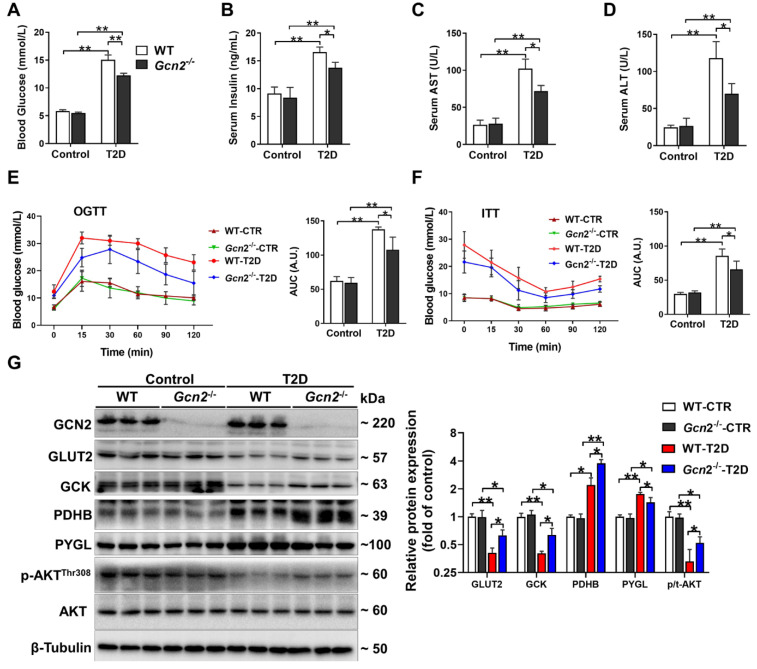
GCN2 deficiency alleviates liver dysfunction, hyperglycemia, and insulin resistance in type 2 diabetic mice. Type 2 diabetes (T2D) was induced in mice with a high-fat diet (HFD) plus low-dose streptozotocin (STZ) injection. At the end of the experiments, fasting blood glucose (**A**), serum insulin (**B**), aspartate transaminase (AST) (**C**), and alanine transaminase (ALT) levels (**D**) were measured. Oral glucose tolerance tests (OGTTs) (**E**) and insulin tolerance tests (ITTs) (**F**) were performed on WT and *Gcn2^−/−^* mice in both the control and T2D groups. The corresponding area under the curve (AUC) values of blood glucose levels in each group were calculated. (**G**) Liver lysates were examined by Western blot. N = 5 (**A**–**F**) or 3 (**G**); values are means ± SD; * indicates *p* < 0.05; ** indicates *p* < 0.01.

To determine the role of GCN2 in T2D, WT and *Gcn2*^−/−^ mice were treated with HFD plus low-dose STZ injection to induce the T2D phenotype. HFD/STZ resulted in significant increases in fasting blood glucose, serum insulin, AST, and ALT levels in both WT and *Gcn2*^−/−^ mice. However, these increases were significantly attenuated in *Gcn2*^−/−^ mice (Figure 1A–D). *Gcn2* deletion did not affect the body weight of control and T2D mice (Figure S1A). The OGTT results showed that the glucose excretion capability was impaired in T2D mice, as evidenced by the area under the curve (AUC) in Figure 1E. However, this impairment in glucose tolerance was significantly attenuated in *Gcn2*^−/−^ mice (Figure 1E). As indicated by the increased AUC in ITTs, GCN2 deficiency also attenuated the decreases in insulin sensitivity in T2D mice (Figure 1F). To investigate the underlying mechanism by which GCN2 affects glucose metabolism, we measured the protein expression of some proteins in the livers of non-diabetic and T2D mice. Western blot results showed that GLUT2, a principal hepatic glucose transporter, and glucokinase (GCK), a kinase that facilitates hepatic glucose uptake during hyperglycemia, were reduced in the livers of T2D mice. However, GCN2 deficiency significantly attenuated the reduction in hepatic GLUT2 and GCK in T2D mice. In addition, pyruvate dehydrogenase beta subunit (PDHB), an enzyme involved in glycolysis, and glycogen phosphorylase (PYGL), a rate-limiting enzyme of glycogenolysis, were upregulated in the livers of T2D mice. GCN2 deficiency promoted the upregulation of PDHB but attenuated the upregulation of PYGL. AKT phosphorylation is tightly associated with insulin signaling and gluconeogenesis [17,18]. In the livers of T2D mice, the phosphorylation of AKT at Thr308 was reduced, and this reduction was attenuated in T2D *Gcn2*^−/−^ mice (Figure 1G).

### 3.2. GCN2 Deficiency Alleviates Hepatic Steatosis and Oxidative Stress in T2D Mice

**Figure 2:**
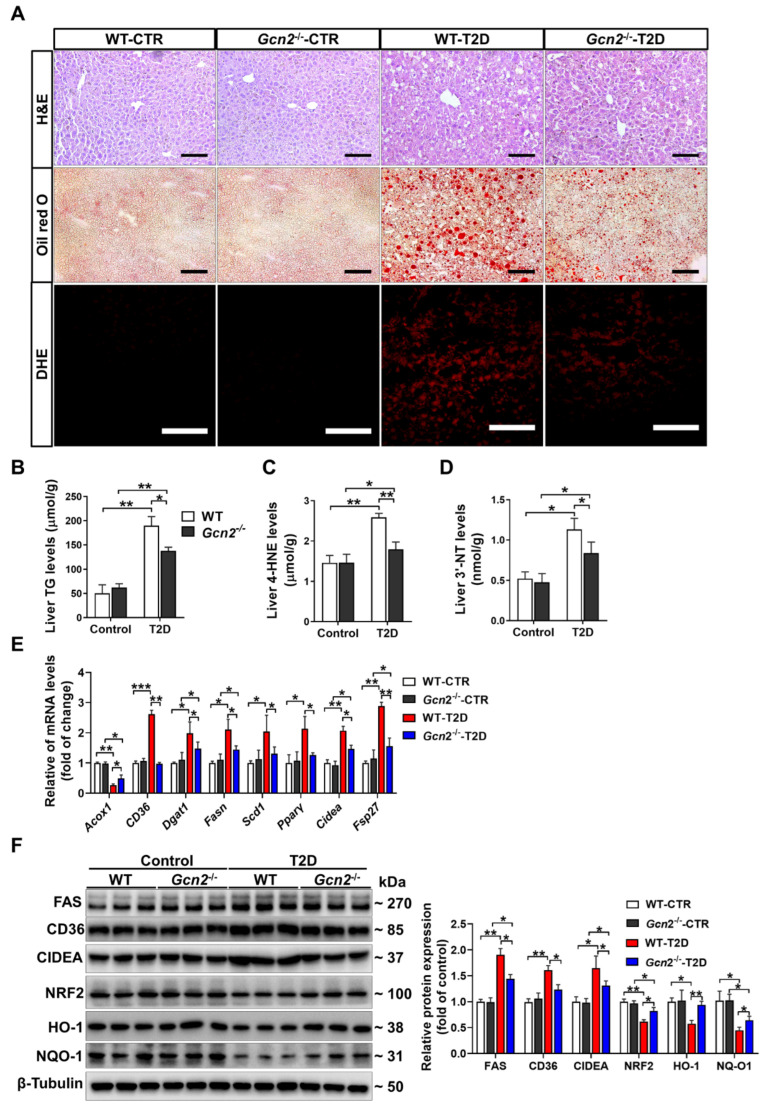
GCN2 deficiency ameliorates hepatic steatosis and oxidative stress in T2D mice. (**A**) Representative liver sections were stained with hematoxylin and eosin (H&E), oil red O, and dihydroethidium (DHE). Scale bar = 100 μm. Liver triglyceride (TG) (**B**), 4-hydroxynonenal (4-HNE) (**C**), and 3′-nitrotyrosine (3′-NT) (**D**) levels were measured. (**E**) The mRNA levels of genes involved in lipid metabolism were measured by qPCR. (**F**) Liver lysates from control and T2D mice were examined by Western blot. N = 5 (**B**–**E**) or 3 (**F**); values are means ± SD; * indicates *p* < 0.05; ** indicates *p* < 0.01; *** indicates *p* < 0.001.

Next, we examined the effect of GCN2 on hepatic lipid metabolism and redox state in T2D mice. As demonstrated by H&E, oil red O, and DHE staining, HFD/STZ treatment resulted in significant histopathological changes, lipid accumulation, and superoxide generation in the livers of T2D mice. However, livers harvested from T2D *Gcn2*^−/−^ mice displayed less hepatic steatosis and lower superoxide levels than those harvested from T2D WT mice (Figure 2A). HFD/STZ treatment also increased hepatic TG, 4-HNE and 3′-NT levels in both genotypes, and these increases were significantly smaller in *Gcn2*^−/−^ mice than in WT mice (Figure 2B–D). In the livers of T2D mice, the mRNA levels of *Acox1* were decreased, and the mRNA levels of *CD36*, *Dgat1*, *Fasn*, *Scd1*, *PPARγ*, *Cidea,* and *Fsp27* were increased. However, the HFD/STZ-induced downregulation of *Acox1* and upregulation of *CD36*, *Dgat1*, *Fasn*, *Scd1*, *PPARγ*, *Cidea,* and *Fsp27* were attenuated by GCN2 deficiency (Figure 2E). Western blotting results demonstrated that GCN2 deficiency attenuated the upregulation of FAS, CD36, and CIDEA and the downregulation of NRF2, HO-1, and NQO-1 in the livers of T2D mice (Figure 2F).

### 3.3. Inhibition of GCN2 Improves Insulin Sensitivity and Alleviates Hepatic Steatosis and Oxidative Stress in T2D Mice

**Figure 3:**
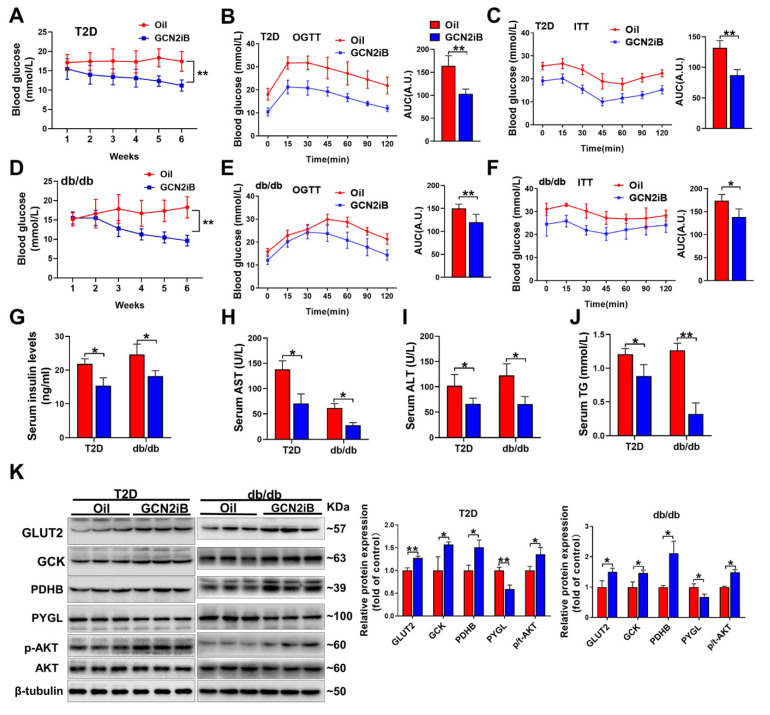
GCN2iB ameliorates hyperglycemia, liver dysfunction, and insulin resistance in T2D mice. HFD plus low-dose STZ injection-induced T2D mice were intraperitoneally injected with oil or GCN2iB (3 mg/kg) every other day for 6 weeks. During the experimental period, fasting blood glucose levels were recorded (**A**). At the end of the experiments, OGTT (**B**) and ITT (**C**) were performed on oil and GCN2iB-treated T2D mice, and the corresponding AUC values of blood glucose levels in each group were calculated. Db/db mice were also treated with the same dose of GCN2iB for 6 weeks, and the changes in fasting blood glucose levels were recorded (**D**). After treatment, OGTT (**E**) and ITT (**F**) were performed, and the corresponding AUCs were calculated. Serum insulin (**G**), AST (**H**), ALT (**I**), and TG (**J**) levels in oil and GCN2iB-treated T2D and db/db mice were measured. (**K**) Western blot analyses were performed on the liver lysates from oil and GCN2iB-treated mice. N = 5 (**A**–**J**) or 3 (**G**); values are means ± SD; * indicates *p* < 0.05; ** indicates *p* < 0.01.

To investigate whether inhibition of GCN2 could be a potential therapeutic approach for T2DM, we used the GCN2-specific inhibitor GCN2iB to treat two T2D mouse models. In HFD/STZ-induced T2D mice, GCN2iB effectively decreased fasting blood glucose levels during the experimental period (Figure 3A). The OGTT and ITT results showed that GCN2iB increased the glucose excretion capability and insulin sensitivity in T2D mice (Figure 3B,C). In db/db mice, GCN2iB also significantly decreased fasting blood glucose levels and improved glucose tolerance and insulin sensitivity (Figure 3D–F). In addition, GCN2iB decreased bodyweight, serum insulin, AST, ALT, and TG levels in both HFD/STZ-induced T2D and db/db mice (Figure 3G–J and Figure S1B). In the livers of the two kinds of T2D mice, GCN2iB treatment resulted in a significant decrease in PYGL protein expression and increases in GLUT2, GCK, and PDHB protein expression and AKT phosphorylation (Figure 3K).

### 3.4. GCN2iB Ameliorates Hepatic Steatosis and Oxidative Stress in T2D Mice

**Figure 4:**
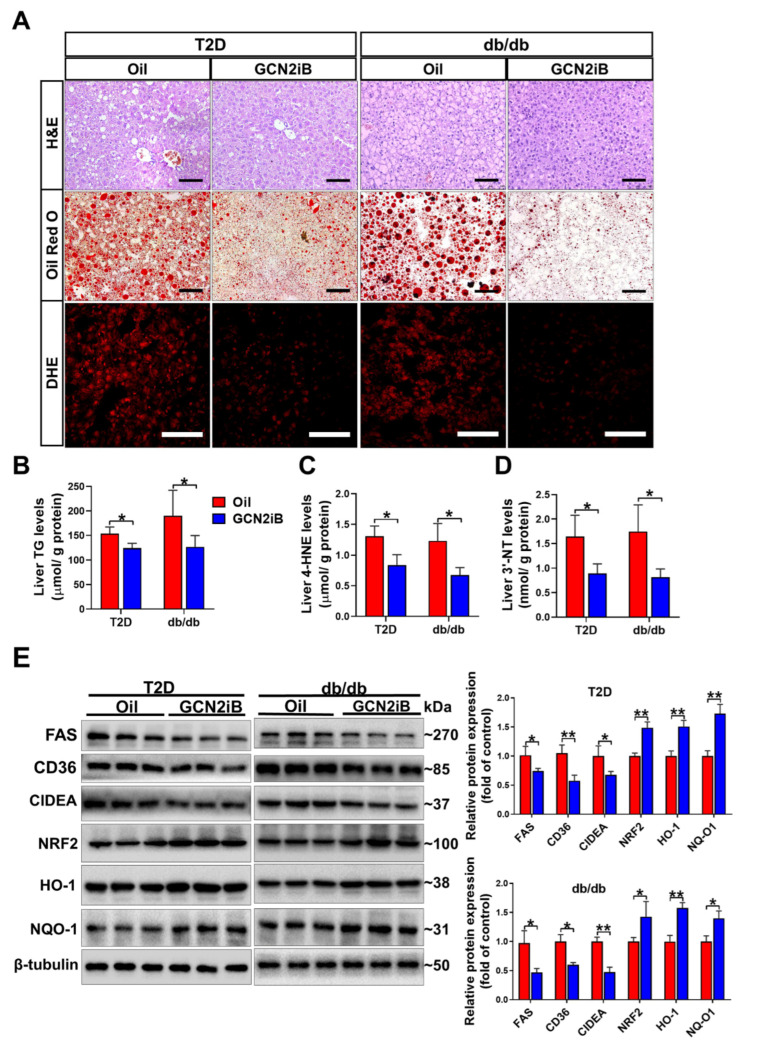
GCN2iB ameliorates hepatic steatosis and oxidative stress in T2D mice. (**A**) Representative liver sections were stained with H&E, oil red O, and DHE. Scale bar = 100 μm. Liver TG (**B**), 4-HNE (**C**), and 3′-NT (**D**) levels were measured. (**E**) Western blot analyses were performed on the liver lysates from oil and GCN2iB-treated mice. N = 5 (**B**–**D**) or 3 (**E**); values are means ± SD; * indicates *p* < 0.05; ** indicates *p* < 0.01.

H&E, oil red O, and DHE staining of liver sections revealed that GCN2iB ameliorated histological abnormalities, hepatic lipid accumulation, and superoxide production in the livers of the two kinds of T2D mice (Figure 4A). GCN2iB treatment also resulted in significant decreases in hepatic TG, 4-HNE, and 3′-NT levels in both HFD/STZ-induced T2D and db/db mice (Figure 4B–D). Western blots showed that GCN2iB caused significant decreases in the protein expression of FAS, CD36, and CIDEA and increases in the protein expression of NRF2, HO-1, and NQO-1 in the livers of T2D mice (Figure 4E).

### 3.5. Depletion of Hepatic Gcn2 Ameliorates Insulin Resistance, Hepatic Steatosis, and Oxidative Stress in db/db Mice

**Figure 5:**
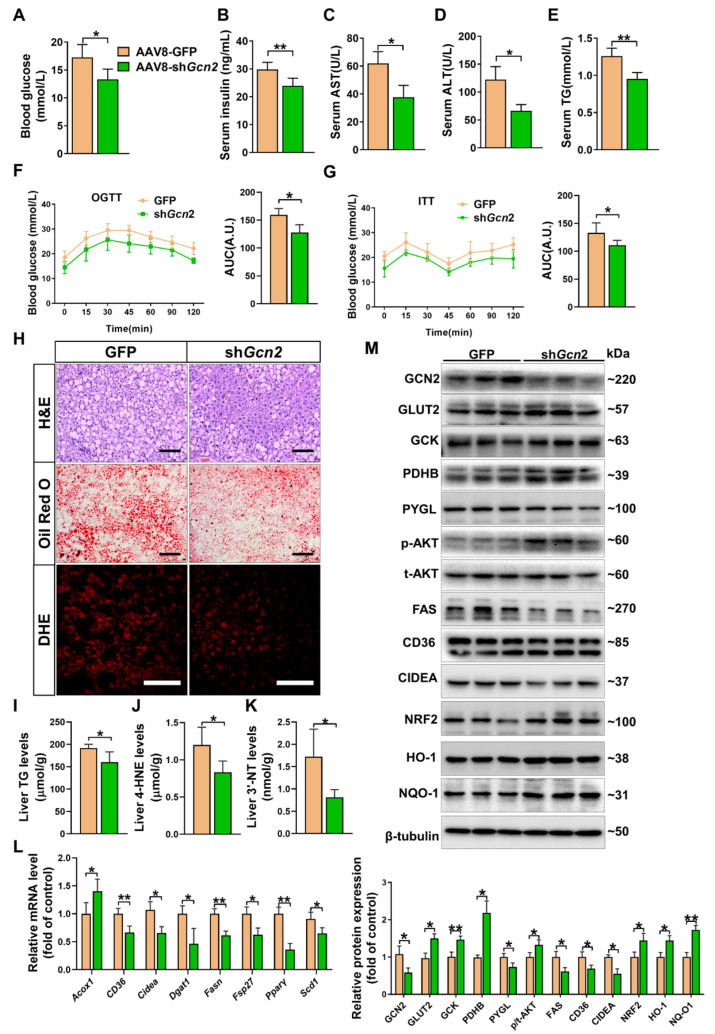
GCN2 knockdown ameliorates metabolic disorders in db/db mice. Db/db mice were treated with AAV8-GFP or AAV8-sh*Gcn2* via intravenous tail injection. After 4 weeks, the mice were sacrificed, and fasting blood glucose (**A**), serum insulin (**B**), AST (**C**), ALT (**D**), and TG (**E**) levels were measured. OGTT (**F**) and ITT (**G**) were performed on GFP- and sh*Gcn2*-treated db/db mice and the corresponding AUC values in each group were calculated. (**H**) Representative liver sections were stained with H&E, oil red O, and DHE. Scale bar = 100 μm. Liver TG) (**I**), 3′-NT (**J**), and 4-HNE (**K**) levels were measured. (**L**) The mRNA levels of lipid metabolic genes were measured by qPCR. (**M**) Liver lysates were analyzed by Western blot. N = 5 (**A**–**L**) or 3 (**M**); values represent the mean ± SD; * indicates *p* < 0.05; ** indicates *p* < 0.01.

To verify whether GCN2 in the liver plays an important role in the development of diabetes, we depleted *Gcn2* from the livers of db/db mice via tail vein injection of AAV8-sh*Gcn2*. Control mice received an AAV8-GFP injection. Knockdown of *Gcn2* decreased fasting blood glucose, serum insulin, AST, ALT, and TG levels (Figure 5A–E) but had no obvious effect on body weight (Figure S1C). The OGTT and ITT results revealed that hepatic *Gcn2* knockdown caused improvements in glucose tolerance and insulin sensitivity in db/db mice (Figure 5F,G). As demonstrated by H&E, oil red O, and DHE staining, *Gcn2* knockdown alleviated liver injury, steatosis, and superoxide generation in db/db mice (Figure 5H). Furthermore, *Gcn2* knockdown decreased liver TG, 4-HNE and 3′-NT levels in the livers of db/db mice (Figure 5I–K). As shown by the qPCR results, the mRNA levels of *Acox1* were increased while the mRNA levels of *CD36*, *Dgat1*, *Fasn*, *Scd1*, *PPARγ*, *Cidea,* and *Fsp27* were decreased in the livers of *Gcn2* depleted db/db mice (Figure 5L). Western blot analysis showed that AAV8-sh*Gcn2* injection caused a ~50% decrease in hepatic GCN2 expression. Moreover, *Gcn2* knockdown resulted in significant increases in the protein expression of GLUT2, GCK, PDHB, p-AKT, NRF2, HO-1, and NQO-1 and decreases in the protein expression of FAS, CD36, and CIDEA (Figure 5M).

## 4. Discussion

The present study has two major findings. First, we demonstrated that genetic and pharmacological inhibition of GCN2 effectively attenuated metabolic disorders in two T2D mouse models. Second, the protective effect of GCN2 inhibition was associated with regulating hepatic glucose and lipid metabolism-related pathways and repression of hepatic oxidative stress.

Although GCN2 is known as a sensor of amino acid availability, it also plays an important role in glucose metabolism by regulating hepatic gluconeogenesis [11] and insulin sensitivity. The present study showed that inhibition of GCN2 in T2D mice by gene deletion or a specific inhibitor resulted in decreases in fasting blood glucose levels and improvements in glucose tolerance and insulin sensitivity. Similar results have also been reported in obese mice [12,14]. Although GCN2 in other cells (e.g., skeletal muscle cells [19], adipocytes [20], and pancreatic β-cells [21]) may have the ability to affect glycemia and whole-body insulin sensitivity in mice. The finding that knockdown of hepatic GCN2 significantly alleviated hyperglycemia and insulin resistance in db/db mice suggested that hepatic GCN2 is an important glycemic regulator in T2D mice.

As the most abundant GLUT isoform in hepatocytes, GLUT2 controls the majority of glucose uptake in the liver but does not affect glucose output [22]. Mutation of GLUT2 increased the risk of T2D in humans [23]. Downregulation of GLUT2 induced by genetic and pharmacological approaches was associated with impaired glucose tolerance and hepatic glucose uptake in mice [24,25]. In addition to GLUT2, GCK maintains glucose homeostasis by regulating glycolysis and hepatic glycogen synthesis. GCK activity and expression were reduced in the livers of diabetic mice [26] and patients with T2D [27,28], while GCK activators are regarded as promising agents for the treatment of T2D [29]. PDHB is the enzyme that catalyzes the conversion of glucose-derived pyruvate to acetyl-CoA and establishes a link between the glycolysis pathway and the tricarboxylic acid cycle (TCA). The present study showed that inhibition of GCN2 increased the protein expression of GLUT2, GCK, and PHDB in the livers of T2D mice, indicating that the reduced glycemia caused by GCN2 inhibition was due, in part, to the promotion of glucose uptake and the glycolysis pathway. In addition, the finding that GCN2 inhibition decreased hepatic PYGL expression suggested that GCN2 might promote hepatic glycogenolysis in T2D. It has been reported that activation of AKT represses hepatic gluconeogenesis through phosphorylation of FoxO1, thereby reducing the expression of PEPCK and G6Pase [17,30]. A previous report showed that global or liver-specific deletion of *Gcn2* failed to induce hepatic gluconeogenesis in fasted mice [11]. Here, we demonstrated that GCN2 inhibition increased the phosphorylation of AKT in the livers of T2D mice, indicating that GCN2 may also affect glycemia by regulating gluconeogenesis.

The strong and complex association between NAFLD and T2D is well recognized. Hyperglycemia and insulin-resistant states in T2D can disrupt the balance between the production and disposal of lipids, which results in increased TG content in the liver [5]. Indeed, liver fat is increased by ~80% in T2D individuals [31]. Here, we also found that both HFD/STZ-induced T2D mice and db/db mice exhibited significant hepatic steatosis, as evidenced by H&E and oil red O staining. In agreement with our previous reports in obese mice [12,14], we demonstrated that GCN2 inhibition alleviated hepatic steatosis in T2D mice, which was associated with upregulation of Acox1 and downregulation of lipid metabolic genes, including *CD36*, *Dgat1*, *Fasn*, *Scd1*, *PPARγ, Cidea,* and *Fsp27*, indicating that GCN2 inhibition repressed lipogenesis and promoted β-oxidation in the livers of T2D mice. Since hepatic lipid accumulation plays an important role in developing whole-body insulin resistance [32], the GCN2 inhibition-mediated alleviation of hepatic steatosis may also be responsible for the increased insulin sensitivity in T2D mice.

Growing evidence suggests that hyperglycemia in T2D can enhance the production of reactive oxygen species (ROS), which leads to excessive oxidative stress [33]. On the other hand, oxidative stress plays a vital role in the development and pathogenesis of T2DM [34]. In recent years, many studies have been carried out to investigate the preventive and healing role of antioxidants in T2D [35]. As a key transcription factor in regulating oxidative stress responses, Nrf2 is a promising target for T2DM therapy. The activators of Nrf2, such as sulforaphane [36,37], curcumin [38,39], resveratrol [40], and TBE-31 [41], significantly increased glucose tolerance and insulin sensitivity in T2D animal models and patients. We recently demonstrated that GCN2 promotes oxidative stress in fatty livers by repressing the Nrf2 signaling pathway. Here, we showed that GCN2 inhibition decreased 4-HNE and 3′-NT levels and increased the protein expression of NRF2, NQO-1, and HO-1 in the livers of T2D mice, indicating that the protective effect of GCN2 inhibition in T2D mice was also associated with activation of the Nrf2 pathway and suppression of hepatic oxidative stress.

Interestingly, our findings differed from a previous report that showed that upregulation of hepatic GCN2 caused by intermittent leucine deprivation or injection of an adenovirus expressing GCN2 improved insulin sensitivity in non-diabetic mice [10]. This discrepancy could be explained as follows: First, although both hyperglycemia and intermittent leucine deprivation can activate/upregulate GCN2, the subsequent response should be fundamentally different. In fact, we previously showed that GCN2 differentially regulates lipogenic genes in response to leucine deprivation and HFD feeding. Second, the vital role of hepatic GCN2 in intermittent leucine deprivation-induced improvement of insulin sensitivity was only observed in non-diabetic mice [10], and GCN2 inhibition experiments in the present study were performed in T2D mice. At any rate, to further address the dual role of GCN2 in T2D therapy, omics technology should be applied to determine the downstream target genes of GCN2 in response to different stimuli. Second, the effect of intermittent leucine deprivation on *Gcn2*^−/−^ T2D mice should be examined.

There are several limitations in the present study. First, the effect of GCN2 inhibition on energy expenditure and physical activity of diabetic mice had not been investigated, which are basic and critically important data to comprehensively evaluate the protective effect of GCN2iB. Second, the response to acute insulin stimulation had not been measured, which is more accurate in evaluating insulin sensitivity.

## 5. Conclusions

In summary, our study indicates that genetic and pharmacological inhibition of GCN2 alleviates hyperglycemia and insulin resistance in T2D mice by regulating hepatic glucose metabolism and attenuating hepatic steatosis and oxidative stress. Our results suggest that GCN2iB administration is a potential approach for T2DM therapy.

## Data Availability

The data presented in this study are available on reasonable request from the corresponding author.

## Supplementary Materials

The following supporting information can be downloaded at: https://www.mdpi.com/article/10.3390/antiox11081584/s1, Table S1: Detail information for primary antibodies; Table S2: The quantitative real-time PCR primer information. Figure S1: Effect of GCN2 inhibition on the body weight of type 2 diabetic mice.
