# Supplementary material for: Genetic and Pharmacological Inhibition of GCN2 Ameliorates Hyperglycemia and Insulin Resistance in Type 2 Diabetic Mice
Source: Antioxidants (Basel). 2022 Aug 16;11(8):1584. doi: 10.3390/antiox11081584 (PMC9404927; doi:10.3390/antiox11081584)
Supplement: Supplementary file 1 [file antioxidants-11-01584-s001.zip › antioxidants-1853494-supplementary.pdf]

Supplemental Files

Genetic and pharmacological inhibition of GCN2 ameliorates hyperglycemia and insulin resistance in type 2 diabetic mice

Juntao Yuan†, Fang Li†, Xiyue Shen†, Junling Gao, Zhuoran Yu, Kai Luo, Bingqing Cui, Zhongbing Lu*

† These authors contributed equally to this work.

* Correspondence

Zhongbing Lu, PhD

E-mail: luzhongbing@ucas.ac.cn

College of Life Science, University of Chinese Academy of Sciences

19A Yuquanlu, Beijing, 100049, China

Fax: 86-10-69672630; Tel: 86-10-69672630

Table S1. Information for antibodies and reagents

Reagent or Resource	Source	Identifier
**Antibodies**		
Rabbit monoclonal [EP1391Y] to Heme Oxygenase 1	Abcam	Cat# ab52947
Mouse monoclonal [A180] to NQO1	Abcam	Cat# ab28947
Rabbit mAb to Fatty Acid Synthase (C20G5)	Cell Signaling Technology	Cat# 3180
Rabbit Polyclonal to GCN2	Cell Signaling Technology	Cat# 3302
Rabbit Polyclonal to p-AKT (Thr308)	Cell Signaling Technology	Cat# 13038
Rabbit Polyclonal to AKT	Cell Signaling Technology	Cat# 9272
Rabbit Polyclonal to β-Tubulin	Cell Signaling Technology	Cat# 2146
Rabbit Polyclonal to NRF2	Proteintech	Cat# 16396-1-AP
Rabbit Polyclonal to GLUT2	Signalway Antibody	Cat# 45050
Rabbit Polyclonal to GCK	Signalway Antibody	Cat# 53975
Rabbit Polyclonal to PDHB	Signalway Antibody	Cat# 30766
Rabbit Polyclonal to PYGL	Signalway Antibody	Cat# 39121
Rabbit polyclonal to CD36	Sino Biological Inc	Cat# 80263-T48
Rabbit polyclonal to Cidea	Sino Biological Inc	Cat# 100879-T32

Table S2. The quantitative real-time PCR primer information

Genes name	Accession	Primers	Sequences (5'-3')	Length (bp)
Acox1	NM_001271898.1	Forward	5′-TGTCTCGCTCCGCTCATAGG -3′	436
		Reverse	5′- ACATGGAGTAATTGAGGCCAACA -3′	
CD36	NM_001159555.1	Forward	5′-CCTGCAAATGTCAGAGGAAA-3′	92
		Reverse	5′-GCGACATGATTAATGGCACA-3′	
Cidea	NM_007702.2	Forward	5′- AGAAGGTCCTACTGACCCCC -3′	266
		Reverse	5′- ACCCGGTGTCCATTTCTGTC -3′	
Dgat1	NM_010046.3	Forward	5′- ATGGACTCTCCAGTTGACGC -3′	318
		Reverse	5′- TCGCACCTCGTCCTCTTCTA -3′	
Fasn	NM_007988.3	Forward	5′- CTCCACAGCTCTTCCAGTGAG -3′	246
		Reverse	5′- TCTCTAGAGGGCTTGCACCA -3′	
Fsp27	NM_001301295.1	Forward	5′- GGGAGGTCCAACACAATCCAA-3′	222
		Reverse	5′- CTCCAAGCTGTGAGCCATGA-3′	
Pparγ	NM_001127330.2	Forward	5′- GCGGAAGAAGAGACCTGGG -3′	116
		Reverse	5′- GTGTGACTTCTCCTCAGCCC -3′	
Scd1	NM_009127.4	Forward	5′- CGCTGGCACATCAACTTCAC-3′	162
		Reverse	5′- AGGAACTCAGAAGCCCAAAGC -3′	
18S	NR_003278.3	Forward	5′-AGGAATTGACGGAAGGGCACCAC-3′	327
		Reverse	5′-GTGCAGCCCCGGACATCTAAGG-3′	

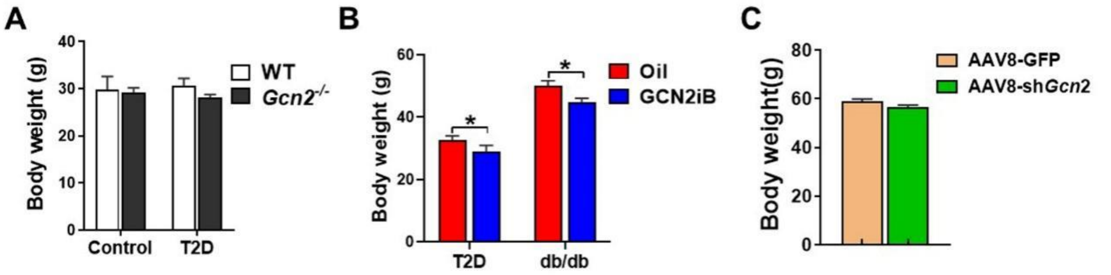

Figure S1. Effect of GCN2 inhibition on bodyweight of type 2 diabetic mice. (**A**) Type 2 diabetes (T2D) was induced in wild type (WT) and *Gcn2*$^{-/-}$ mice with a high-fat diet (HFD) plus low-dose strepto-zotocin (STZ) injection. At the end of the experiments, body weight was recorded. (**B**) After treatment with oil or GCN2iB (3 mg/kg, every other day) for 6 weeks, bodyweight of HFD plus low-dose STZ injection-induced T2D mice and db/db mice were recorded. (**C**) After tail intravenous injection of AAV8-GFP or AAV8-sh*Gcn2* for 4 weeks, the bodyweight of db/db mice were recorded. N=5; values represent the mean ± SD; * indicates $p<0.05$.
